# Aberrant regulation of LncRNA TUG1-microRNA-328-3p-SRSF9 mRNA Axis in hepatocellular carcinoma: a promising target for prognosis and therapy

**DOI:** 10.1186/s12943-021-01493-6

**Published:** 2022-02-04

**Authors:** Yudong Liu, Xia Mao, Zhaochen Ma, Wenjia Chen, Xiaodong Guo, Lingxiang Yu, Xinxin Deng, Funeng Jiang, Taixian Li, Na Lin, Yanqiong Zhang

**Affiliations:** 1 Institute of Chinese Materia Medica, China Academy of Chinese Medical Sciences, Beijing, 100700 China; 2 Fifth Medical Center of PLA General Hospital, Beijing, 100039 China

Hepatocellular carcinoma (HCC), representing the dominant type of liver cancer, is responsible for the great percentage of total diagnoses and deaths [1]. Its incidence with heterogeneous property has been increasing in high-rate areas and decreasing in many low-rate areas, due to the geographic variations for the underlying risk factors, including the prevalence of hepatitis B/C virus, the consumption level of alcohol, and the morbidity of metabolic syndrome and nonalcoholic fatty liver disease, etc [2]. Despite the optimization of diagnostic and therapeutic strategy for HCC treatment, the prognosis has not been markedly improved [3]. Therefore, elucidating tumor-specific mechanisms for HCC onset and progression is essential for identifying novel therapeutic targets and developing therapeutic strategies.

Accumulating evidence has indicated the vital role of noncoding RNAs (ncRNAs) in HCC carcinogenesis. LncRNAs in the cytoplasm regulate protein levels, either by directly maintaining mRNA stability or by acting as competing endogenous RNA (ceRNA) [4]. In 2011, Salmena et al. put forward a ceRNA hypothesis, that is, mRNAs, lncRNAs and other ncRNAs share common miRNA response elements (MREs), thus restraining miRNA biological function by competitively binding with MREs on the target mRNA [5]. Therefore, lncRNAs and mRNAs may act as potential diagnostic and prognostic biomarkers for HCC due to their good specificity and accessibility. In the current study, a novel lncRNA TUG1-microRNA-328-3p-SRSF9 mRNA axis involved in HCC carcinogenesis, was identified, thus providing meaningful clues for the targeted prognosis and therapy of HCC.

## Results and discussion

### Dysregulation of lncRNA TUG1-miR-328-3p-SRSF9 mRNA axis associates with aggressive progression and patients’ prognosis of HCC

To identify the transcripts that were involved in hepatocarcinogenesis, lncRNA, miRNA and mRNA expression profiles were detected in discovery cohort using microarray analysis (GSE166163). Approximately 1169 lncRNAs, 89 miRNAs and 1766 mRNAs were differentially expressed in HCC tissues compared with those in the adjacent non-cancerous liver tissues. Among them, hsa-miR-328-3p expression was significantly down-regulated in HCC tissues compared with the adjacent non-cancerous liver tissues, and highly expressed SRSF9 mRNA was predicted to be one of the candidate targets for hsa-miR-328-3p. Then, lncRNA TUG1 binding the same 3′-UTR site of hsa-miR-328-3p with SRSF9 mRNA was screened by miRanda, and its expression trend in HCC tissues and the adjacent non-cancerous liver tissues was also similar to that of SRSF9 mRNA. Moreover, the dual-luciferase reporter gene assay validated that both SRSF9 mRNA and lncRNA TUG1 were the targets of hsa-miR-328-3p with the same binding site (Additional file 1: Fig. S1).

**Fig. 1:**
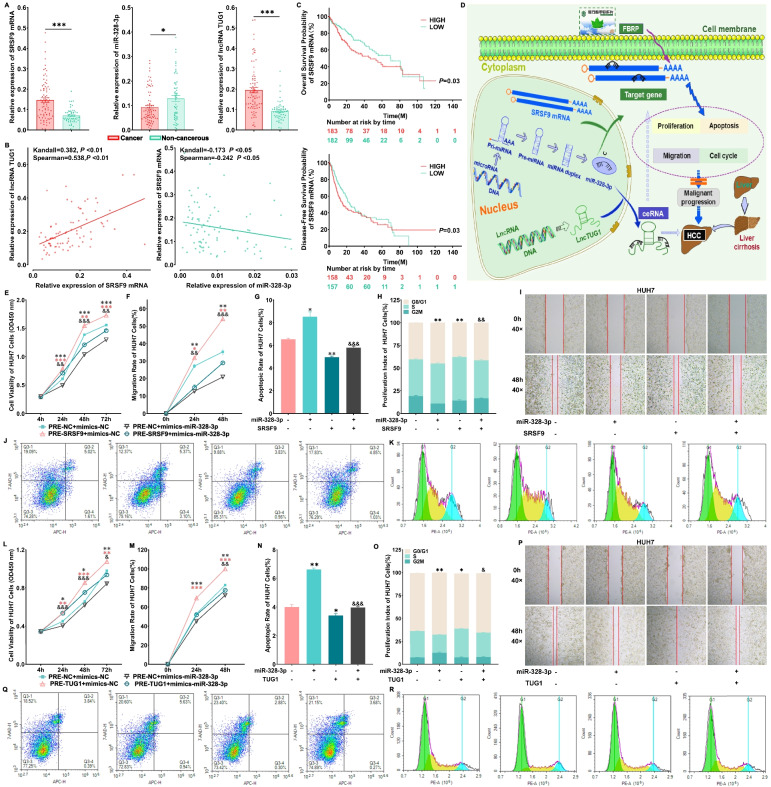
Dysregulation of lncRNA TUG1-miR-328-3p-SRSF9 mRNA ceRNA axis was involved in the hepatocarcinogenesis. **A** Relative expression levels of SRSF9 mRNA, miR-328-3p and lncRNA TUG1 in HCC tissues (*n* = 77) compared with the corresponding non-cancerous liver tissues (*n *= 56). *p < 0.05, **p < 0.01, ***p < 0.001, comparison with the non-cancerous group. **B **Kandall and Spearman correlation analyses between miR-328-3p and SRSF9 mRNA expression levels, as well as between SRSF9 mRNA and lncRNA TUG1 expression levels in HCC samples. **C** SRSF9 mRNA expression may be significantly associated with the overall survival rate and disease-free survival rate of HCC patients. **D **The schematic model of the lncRNA TUG1-miR-328-3p-SRSF9 mRNA axis involved into the progression of HCC. **E, F, H, I.** SRSF9 overexpression reverses the tumor suppressive roles of miR-328-3p in the proliferation and migration activities in HUH7 cells. CCK-8 assays were carried out to determine the cell viability for pre-SRSF9 mRNA+mimics-miR-328-3p transfected HUH7 cells. Wound Healing assays were carried out to determine the cell mobility for pre-SRSF9 mRNA+mimics-miR-328-3p transfected HUH7 cells. **G, J, K **Apoptosis and cell cycle were determined by flow cytometry for pre-SRSF9 mRNA + mimics-miR-328-3p transfected HUH7 cells. Data were represented as the mean ± sem. From three independent experiments. ^*^p < 0.05, ^**^p < 0.01, ^***^p < 0.001, comparison with the pre-NC + mimics-NC group; ^&^*p* < 0.05, ^&&^*p* < 0.01, ^&&&^*p* < 0.001, comparison with the pre-NC + mimics-miR-328-3p group. ^#^*p* < 0.05, ^##^*p* < 0.01, ^###^*p* < 0.001, comparison with the pre-NC + pre-SRSF9 group. **L, M, O, P **LncRNA TUG1 competitively disturbs the regulatory effects of miR-328-3p in cell proliferation and migration in HUH7 cells. CCK-8 assays were carried out to determine the cell viability for pre-lncRNA TUG1 + mimics-miR-328-3p transfected HUH7 cells. Wound Healing assays were carried out to determine the cell mobility for pre-lncRNA TUG1 + mimics-miR-328-3p transfected HUH7 cells. **N, Q, R **Apoptosis and cell cycle were determined by flow cytometry for pre-lncRNA TUG1 + mimics-miR-328-3p transfected HUH7 cells. Data were represented as the mean ± sem. From three independent experiments. ^*^p < 0.05, ^**^p < 0.01, ^***^p < 0.001, comparison with the pre-NC + mimics-NC group; ^&^p < 0.05, ^&&^p < 0.01, ^&&&^p < 0.001, comparison with the pre-NC + mimics-miR-328-3p group. ^#^p < 0.05, ^##^p < 0.01, ^###^p < 0.001, comparison with the pre-NC + pre-lncRNA TUG1 group

To confirm the aberrant expression patterns of lncRNA TUG1, miR-328-3p and SRSF9 mRNA in HCC, real-time qPCR was performed based on the validation cohort, the results of which was in line with the microarray data based on the discovery cohort (Fig. 1A). In addition, the significant correlations between miR-328-3p and SRSF9 mRNA expression levels, as well as SRSF9 mRNA and lncRNA TUG1 expression levels were observed in HCC samples according to both Kandall and Spearman analyses (Fig. 1B).

The clinical significance of SRSF9 mRNA and lncRNA TUG1 expression in HCC was evaluated based on the TCGA data and our cohorts. As a result, the occurrence of SRSF9 mRNA and lncRNA TUG1 overexpression in male HCC patients was both more frequent than that in female. High expression of SRSF9 mRNA and lncRNA TUG1 were significantly associated with preoperative serum alpha-fetoprotein (AFP) level and high tumor grade. Importantly, Kaplan-Meier survival curve analysis indicated that HCC patients with high SRSF9 mRNA expression had worse overall and disease-free survivals than those with low expression (both *P* < 0.05) (Fig. 1C). However, multivariate cox regression analysis of lncRNA TUG1 and SRSF9 mRNA based on TCGA database indicated that both lncRNA TUG1 and SRSF9 mRNA expression were not independent prognostic factors in HCC (no expression data of microRNA-328-3p in TCGA database) (Additional file 2: Table S1 ~ S2), and there is no significant correlation between lncRNA TUG1 and SRSF9 mRNA expression according to the TCGA data (Additional file 3: Table S3).

### Suppression of SRSF9 mRNA and up-regulation of miR-328-3p both efficiently inhibit HCC cell proliferation, migration, cell cycle, and induce HCC cell apoptosis

To gain insight into the biological function of SRSF9 mRNA and miR-328-3p in HCC cellular malignancy, specific siRNAs against SRSF9 mRNA gene transcript (Additional file 4: Fig. S2A ~ B), and plasmids that stably up-regulated the endogenous expression of miR-328-3p were successfully imported into HUH7 and MHCC97H cells, respectively. Results showed that both low expression of SRSF9 mRNA and up-regulation of miR-328-3p significantly repressed the ability of cell proliferation and reduced cell capacity of migration of HCC cells. Besides, they led to cell cycle arrest in G1 phase and markedly increased the percentage of apoptotic HCC cells (Additional files 5, 6: Fig. S3, S4).

### SRSF9 restoration reverses the tumor suppressive effects of miR-328-3p in HCC cells

To verify the regulatory effects of miR-328-3p on SRSF9 mRNA in exerting its anti-HCC effects in vitro, miR-328-3p and SRSF9 mRNA co-overexpression plasmids were constructed and imported into HUH7 and MHCC97H cells, respectively. Following that, the expression levels of SRSF9 protein in HCC cells imported with miR-328-3p and SRSF9 mRNA co-overexpression plasmids were markedly higher than those with miR-328-3p overexpression plasmid alone (Additional file 7: Fig. S5A, C). Functionally, compared with PRE-NC + mimics-NC group, up-regulation of miR-328-3p alone in HUH7 and MHCC97H cells resulted in increasing proliferation abilities and HCC cells apoptosis, as well as diminished migratory potential and percentage of cells in S and G2 phases. Conversely, a notable reverse trend of these parameters was obtained by the co-transfection of miR-328-3p and SRSF9 mRNA in HUH7 and MHCC97H cells, analyzed via CCK-8 assay, wound healing assay and flow cytometry assay (Fig. 1E~K, Additional file 8: Fig. S6A ~ G).

### LncRNA TUG1 acts as an endogenous “sponge” by binding miR-328-3p, and thus abolishes miRNA-328-3p’s negative regulation on SRSF9 mRNA

To further verify the effects of lncRNA TUG1 in influencing the intervention of miR-328-3p on SRSF9 mRNA in HCC, the plasmids that overexpressed miR-328-3p and lncRNA TUG1 were co-transfected into HUH7 and MHCC97H cells, the expression levels of SRSF9 protein of which were markedly higher than those with miR-328-3p overexpression plasmid alone (Additional file 7: Fig. S5B, D), implying that lncRNA TUG1 might notably reduce the negatively regulatory effect of miRNA-328-3p on the expression of SRSF9 in HCC cell lines. Similar to the findings of miR-328-3p and SRSF9 mRNA co-overexpression group, the co-overexpression of miR-328-3p and lncRNA TUG1 significantly attenuated the tumor suppressive roles of miR-328-3p in HCC cells (Fig. 1L~R, Additional file 8: Fig. S6H ~ N).

### lncRNA TUG1-miR-328-3p-SRSF9 mRNA axis may be involved into tumor growth in vivo and effectively reduced by the anti-HCC prescription FBRP

**Fig. 2:**
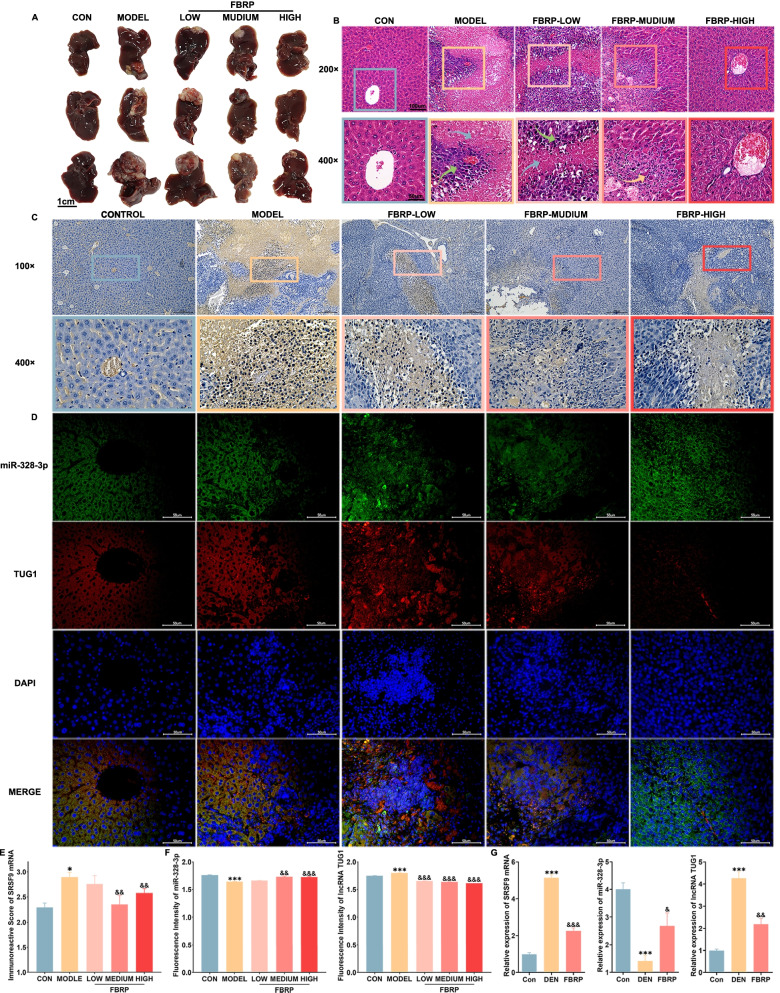
LncRNA TUG1-miR-328-3p-SRSF9 mRNA axis may be involved into tumor growth in vivo and may be effectively regulated by the anti-HCC prescription FBRP.** A **Pharmacological effects of FBRP on pathological changes into the liver tissues of the nude mice with carcinoma in situ.** B **Pharmacological effects of FBRP on the histological changes into liver tissues of the in subcutaneous xenograft HCC nude mouse model. Boxes represent the area with typical pathological changes. Green arrows represent the HCC cells. Red arrows represent the necrotic cells among HCC cells. Blue arrows represent massive infiltration of inflammatory cells. **C **Generation of neoplastic lesions of nude mice in different groups.** D, E, F **Expression and subcellular localization of SRSF9 protein in liver tissues of nude mice and the quantification results examined by immunohistochemistry. Quantification, localization and co-localization of miR-328-3p and lncRNA TUG1 were detected by RNA FISH assay. Data were presented as the mean ± sem. From three independent experiments. ^*^p < 0.05, ^**^p < 0.01, ^***^p < 0.001, comparison with the CON group; ^&^p < 0.05, ^&&^p < 0.01, ^&&&^p < 0.001, comparison with the model group. **G **The expression levels of SRSF9 mRNA, miR-328-3p and lncRNA TUG1 in liver tissues of DEN-induced “Hepatitis-Liver Fibrosis-Liver Cancer” malignant transformation rats and FBRP intervention rats. Data were presented as the mean ± sem. From three independent experiments. ^*^p < 0.05, ^**^p < 0.01, ^***^p < 0.001, comparison with the CON group; ^&^p < 0.05, ^&&^p < 0.01, ^&&&^p < 0.001, comparison with the DEN group

To investigate the role of lncRNA TUG1-miR-328-3p-SRSF9 mRNA ceRNA axis in vivo, a stable DEN-induced rat model presenting the progression of hepatic fibrosis-cirrhosis-cancer was constructed as described in our previous study [6], and a subcutaneous xenograft HCC nude mouse model was also successfully constructed to simulate the hepatocarcinogenesis. Results showed the body weight and relative liver weight of nude mice in model group decreased and then changed in a fluctuation, presenting a decreasing trend when compared with normal control group (Additional files 9, 10: Fig. S7, S8, Fig. 2A). Pathological changes were observed in nude mice of the model group after HCC induction (Fig. 2B). The decreased expression of miR-328-3p, and the enhanced expression of lncRNA TUG1/SRSF9 mRNA in nude mice of model group were observed when compared with normal control group by immunohistochemistry and RNA FISH assay (Fig. 2C, D, E, F), in line with the real-time qPCR analysis based on DEN models (Fig. 2G), all of which can be recovered by the treatment of Fufang Biejia Ruangan Pill (FBRP), that has been proved to notably improve fibrosis progression and prevent the generation of HCC (Fig. 2) [6].

## Conclusions

Our data offer the evidence that the lncRNA TUG1-miR-328-3p-SRSF9 mRNA axis function as a novel ceRNA regulatory axis, which may be associated with HCC malignancy and may be one of therapeutic targets of the anti-HCC prescription FBRP (Fig. 1D). These findings may improve our understanding of molecular mechanisms involved in the aggressive progression of HCC, and may highlight a promising target for the prognosis and treatment of this malignancy.

## Supplementary Information

**Additional file 1: Figure S1. **Dual-luciferase reporter gene assay based on 293 T cells was performed to verify whether SRSF9 mRNA and lncRNA TUG1 were the targets of hsa-miR-328-3p with the same binding site. Genetic sequence marked with the rectangle indicated the same binding site that shared by SRSF9 mRNA and lncRNA TUG1 with miR-328-3p. ^*^p < 0.05, ^**^p < 0.01, ^***^p < 0.001, comparison with the miR-328-3p + WT-SRSF9 mRNA (A) or lncRNA TUG1 (B) groups.

**Additional file 2: Table S1 ~ S2.** Multivariate cox regression analysis on various clinicopathological features of HCC patients with SRSF9 mRNA and lncRNA TUG1 expression data.

**Additional file 3: Table S3.** Analysis for the positive correlation of lncRNA TUG1 and SRSF9 mRNA in TCGA cohort (with the different etiology)

**Additional file 4: Figure S2.** Screening of specific siRNA against SRSF9 mRNA gene transcript that most efficiently down-regulated the expression of SRSF9 mRNA in HUH7 and MHCC97H cells. ^*^p < 0.05, ^**^p < 0.01, ^***^p < 0.001, comparison with the si-NC group.

**Additional file 5: Figure S3**. Suppression of SRSF9 mRNA significantly inhibits HCC cell proliferation, migration, cell cycle, and promotes HCC cell apoptosis. CCK-8 assays were used to determine the cell viability for si-SRSF9 transfected HUH7 (A) and MHCC97H (C) cells. Wound Healing assays were used to determine the cell capacity of migration for si-SRSF9 transfected HUH7 (B, I) and MHCC97H (D, J) cells. Apoptosis and cell cycle was determined by flow cytometry for si-SRSF9 mRNA transfected HUH7 (E, F, K, M) and MHCC97H (G, H, L, N) cells. Data were represented as the mean ± sem. From three independent experiments. ^*^p < 0.05, ^**^p < 0.01, ^***^p < 0.001, comparison with the si-NC group.

**Additional file 6: Figure S4. **Enforced expression of miR-328-3p efficiently inhibits HCC cell proliferation, migration, cell cycle, and promotes HCC cell apoptosis. CCK-8 assays were used to determine the cell viability for mimics-miR-328-3p transfected HUH7 (A) and MHCC97H (C) cells. Wound Healing assays were used to determine the cell ability of migration for mimics-miR-328-3p transfected HUH7 (B, I) and MHCC97H (D, J) cells. Apoptosis and cell cycle were determined by flow cytometry for mimics-miR-328-3p transfected HUH7 (E, F, K, M) and MHCC97H (G, H, L, N) cells. Data were represented as the mean ± sem. From three independent experiments. ^*^p < 0.05, ^**^p < 0.01, ^***^p < 0.001, comparison with the mimics-NC group.

**Additional file 7: Figure S5. **The expression levels of SRSF9 protein in (A, B) HUH7, (C, D) MHCC97H cells that were imported with miR-328-3p and SRSF9 co-overexpression plasmids. Data were represented as the mean ± sem. From three independent experiments. ^*^p < 0.05, ^**^p < 0.01, ^***^p < 0.001, comparison with the pre-NC + mimics-NC group; ^&^p < 0.05, ^&&^p < 0.01, ^&&&^p < 0.001, comparison with the pre-NC + mimics-miR-328-3p group. ^#^p < 0.05, ^##^p < 0.01, ^###^p < 0.001, comparison with the pre-NC + pre-lncRNA TUG1/SRSF9 mRNA group.

**Additional file 8: Figure S6. **Functional analysis of miR-328-3p alone, the co-transfection of miR-328-3p and SRSF9 mRNA, and the co-overexpression of miR-328-3p and lncRNA TUG1 in MHCC97H cells, analyzed via CCK-8 assay, wound healing assay and flow cytometry assay. A, B, D, E. SRSF9 overexpression reverses the tumor suppressive roles of miR-328-3p in the proliferation and migration activity in MHCC97H cells. CCK-8 assays were carried out to determine the cell viability for pre-SRSF9 mRNA+mimics-miR-328-3p transfected MHCC97H cells. Wound Healing assays were carried out to determine the cell mobility for pre-SRSF9 mRNA+mimics-miR-328-3p transfected MHCC97H cells. C, F, G. Apoptosis and cell cycle were determined by flow cytometry for pre-SRSF9 mRNA + mimics-miR-328-3p transfected MHCC97H cells. Data were represented as the mean ± sem. From three independent experiments. ^*^p < 0.05, ^**^p < 0.01, ^***^p < 0.001, comparison with the pre-NC + mimics-NC group; ^&^p < 0.05, ^&&^p < 0.01, ^&&&^p < 0.001, comparison with the pre-NC + mimics-miR-328-3p group. ^#^p < 0.05, ^##^p < 0.01, ^###^p < 0.001, comparison with the pre-NC + pre-SRSF9 group. H, I, K, L. LncRNA TUG1 competitively disturbs the regulatory effects of miR-328-3p in cell proliferation and migration in MHCC97H cells. CCK-8 assays were carried out to determine the cell viability for pre-lncRNA TUG1 + mimics-miR-328-3p transfected MHCC97H cells. Wound Healing assays were carried out to determine the cell mobility for pre-lncRNA TUG1 + mimics-miR-328-3p transfected MHCC97H cells. J, M, N. Apoptosis and cell cycle were determined by flow cytometry for pre-lncRNA TUG1 + mimics-miR-328-3p transfected MHCC97H cells. Data were represented as the mean ± sem. From three independent experiments. ^*^p < 0.05, ^**^p < 0.01, ^***^p < 0.001, comparison with the pre-NC + mimics-NC group; ^&^p < 0.05, ^&&^p < 0.01, ^&&&^p < 0.001, comparison with the pre-NC + mimics-miR-328-3p group. ^#^p < 0.05, ^##^p < 0.01, ^###^p < 0.001, comparison with the pre-NC + pre-lncRNA TUG1 group.

**Additional file 9: Figure S7. **Changes of the body weight of subcutaneous xenograft HCC nude mouse in control, model and FBRP treatment groups at different time points during experiments. ^*^p < 0.05, ^**^p < 0.01, ^***^p < 0.001, comparison with groups marked in corresponding color.

**Additional file 10: Figure S8.** Liver index of subcutaneous xenograft HCC nude mouse in control, model and FBRP treatment groups at different time points during experiments.

**Additional file 11.** Materials and methods

## Data Availability

Datasets used and/or analyzed during the current study are available from the corresponding author on reasonable request.

## Abbreviations

ceRNA: competing endogenous RNA
FISH: Fluorescence In Situ Hybridization
FBRP: Fufang Biejia Ruangan Pill
HCC: Hepatocellular Carcinoma
lncRNA: Long non-coding RNAs
miRNA: microRNA
MREs: miRNA Response Elements
ncRNAs: noncoding RNAs
SRSF9: Serine-arginine-Rich Splicing Factor 9
siRNA: small interfering RNA
TUG1: Taurine Up-regulated1
TCGA: The Cancer Genome Atlas
