# Supplementary material for: Aberrant regulation of LncRNA TUG1-microRNA-328-3p-SRSF9 mRNA Axis in hepatocellular carcinoma: a promising target for prognosis and therapy
Source: Mol Cancer. 2022 Feb 4;21:36. doi: 10.1186/s12943-021-01493-6 (PMC8815183; doi:10.1186/s12943-021-01493-6)
Supplement: Supplementary file 1 — Additional file 1: Figure S1. Dual-luciferase reporter gene assay based on 293 T cells was performed to verify whether SRSF9 mRNA and lncRNA TUG1 were the targets of hsa-miR-328-3p with the same binding site. Genetic sequence marked with the rectangle indicated the same binding site that shared by SRSF9 mRNA and lncRNA TUG1 with miR-328-3p. *p < 0.05, **p < 0.01, ***p < 0.001, comparison with the miR-328-3p + WT-SRSF9 mRNA (A) or lncRNA TUG1 (B) groups. [file 12943_2021_1493_MOESM1_ESM.docx]

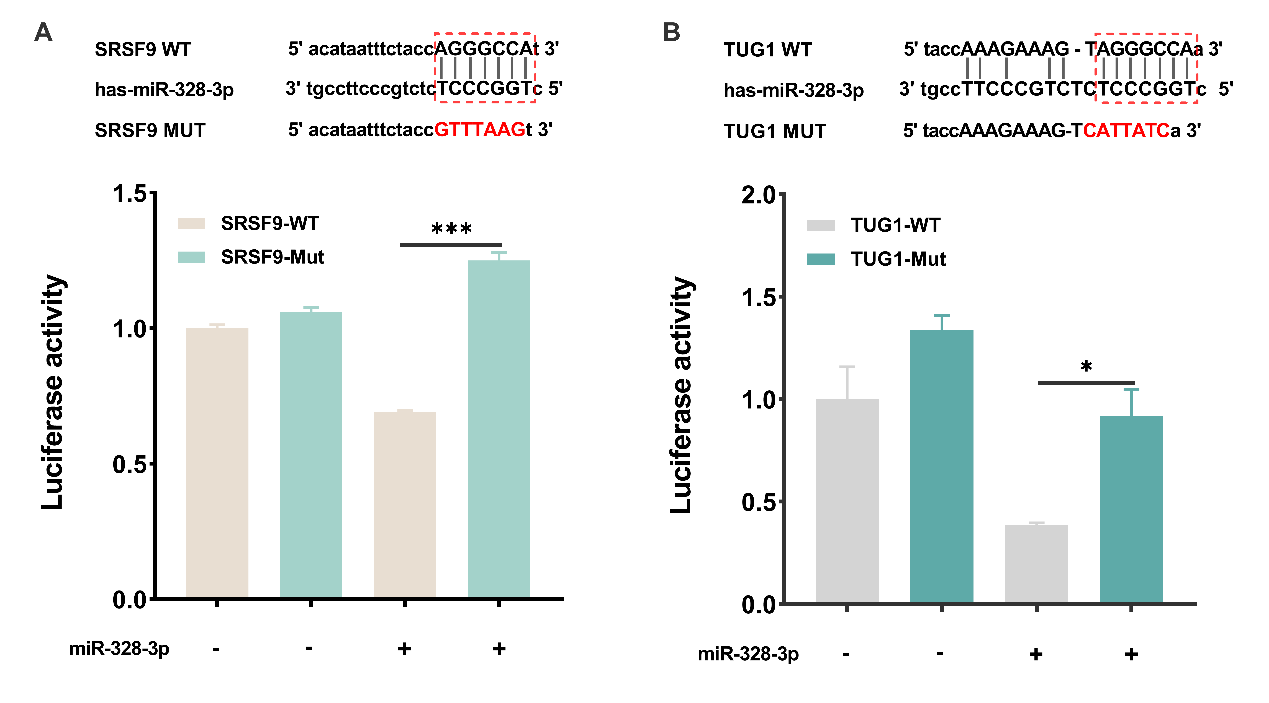
**Additional file 1: Figure S1. Dual-luciferase reporter gene assay based on 293T cells was performed to verify whether SRSF9 mRNA and lncRNA TUG1 were the targets of hsa-miR-328-3p with the same binding site. Genetic sequence marked with the rectangle indicated the same binding site that shared by SRSF9 mRNA and lncRNA TUG1 with miR-328-3p.** ^*^p < 0.05, ^**^p < 0.01, ^***^p < 0.001, comparison with the miR-328-3p +WT-SRSF9 mRNA (A) or lncRNA TUG1 (B) groups.
