# Supplementary material for: Aberrant regulation of LncRNA TUG1-microRNA-328-3p-SRSF9 mRNA Axis in hepatocellular carcinoma: a promising target for prognosis and therapy
Source: Mol Cancer. 2022 Feb 4;21:36. doi: 10.1186/s12943-021-01493-6 (PMC8815183; doi:10.1186/s12943-021-01493-6)
Supplement: Supplementary file 2 — Additional file 2: Table S1 ~ S2. Multivariate cox regression analysis on various clinicopathological features of HCC patients with SRSF9 mRNA and lncRNA TUG1 expression data. [file 12943_2021_1493_MOESM2_ESM.docx]

**Additional file 2: Multivariate cox regression analysis on various clinicopathological features of HCC patients with SRSF9 mRNA and lncRNA TUG1 expression data**

| **Additional file 2: Table S1. Multivariate cox regression analysis on various clinicopathological features of HCC patients with SRSF9 mRNA expression data** | | | | | | |
| --- | --- | --- | --- | --- | --- | --- |
|  | **B** | **SE** | **Wald** | **df** | **sig** | **Exp(B)** |
| **SRSF9 mRNA expression** | 0.093 | 0.281 | 0.111 | 1 | 0.740 | 1.098 |
| **AGE** | 0.506 | 0.271 | 3.498 | 1 | 0.061 | 1.659 |
| **Sex** | 0.078 | 0.311 | 0.062 | 1 | 0.803 | 1.081 |
| **Preoperative serum AFP level** | 0.201 | 0.291 | 0.480 | 1 | 0.488 | 1.223 |
| **Liver fibrosis ishak score category** | -0.141 | 0.309 | 0.208 | 1 | 0.648 | 0.868 |
| **Neoplasm Histologic Grade** | 0.425 | 0.202 | 4.414 | 1 | 0.036 | 1.529 |
| **American Joint Committee on Cancer Tumor Stage Code** | 0.414 | 0.144 | 8.241 | 1 | 0.004 | 1.513 |

**Additional file 2: Table S2. Multivariate cox regression analysis on various clinicopathological features of HCC patients with lncRNA TUG1 expression data**

|  | **B** | **SE** | **Wald** | **df** | **sig** | **Exp(B)** |
| --- | --- | --- | --- | --- | --- | --- |
| **LncRNA TUG1 expression** | -1.039 | 1.027 | 1.022 | 1 | 0.312 | 0.354 |
| **AGE** | 0.079 | 0.204 | 0.151 | 1 | 0.697 | 1.083 |
| **Sex** | -0.042 | 0.246 | 0.029 | 1 | 0.866 | 0.959 |
| **Preoperative serum AFP level** | 0.440 | 0.216 | 4.126 | 1 | 0.042 | 1.552 |
| **Liver fibrosis ishak score category** | 0.321 | 0.248 | 1.669 | 1 | 0.196 | 1.378 |
| **Neoplasm Histologic Grade** | 0.151 | 0.155 | 0.948 | 1 | 0.330 | 1.162 |
| **American Joint Committee on Cancer Tumor Stage Code** | 0.446 | 0.109 | 16.645 | 1 | <0.001 | 1.563 |
