# Supplementary material for: Aberrant regulation of LncRNA TUG1-microRNA-328-3p-SRSF9 mRNA Axis in hepatocellular carcinoma: a promising target for prognosis and therapy
Source: Mol Cancer. 2022 Feb 4;21:36. doi: 10.1186/s12943-021-01493-6 (PMC8815183; doi:10.1186/s12943-021-01493-6)
Supplement: Supplementary file 3 — Additional file 3: Table S3. Analysis for the positive correlation of lncRNA TUG1 and SRSF9 mRNA in TCGA cohort (with the different etiology) [file 12943_2021_1493_MOESM3_ESM.docx]

**Additional file 3: Table S3. Analysis for the positive correlation of lncRNA TUG1 and SRSF9 mRNA in TCGA cohort (with the different etiology)**

| Positive correlation analysis of lncRNA TUG1 and SRSF9 mRNA in TCGA cohort | | | | |
| --- | --- | --- | --- | --- |
|  | | | SRSF9 | TUG1 |
| Kandall | SRSF9 | Correlation coefficient | 1.000 | 0.014 |
|  |  | Sig. |  | 0.684 |
|  |  | N | 373 | 370 |
|  | TUG1 | Correlation coefficient | 0.014 | 1.000 |
|  |  | Sig. | 0.684 |  |
|  |  | N | 370 | 370 |
| Spearman | SRSF9 | Correlation coefficient | 1.000 | 0.025 |
|  |  | Sig. |  | 0.632 |
|  |  | N | 373 | 370 |
|  | TUG1 | Correlation coefficient | 0.025 | 1.000 |
|  |  | Sig. | 0.632 |  |
|  |  | N | 370 | 370 |
