# Supplementary material for: Aberrant regulation of LncRNA TUG1-microRNA-328-3p-SRSF9 mRNA Axis in hepatocellular carcinoma: a promising target for prognosis and therapy
Source: Mol Cancer. 2022 Feb 4;21:36. doi: 10.1186/s12943-021-01493-6 (PMC8815183; doi:10.1186/s12943-021-01493-6)
Supplement: Supplementary file 5 — Additional file 5: Figure S3. Suppression of SRSF9 mRNA significantly inhibits HCC cell proliferation, migration, cell cycle, and promotes HCC cell apoptosis. CCK-8 assays were used to determine the cell viability for si-SRSF9 transfected HUH7 (A) and MHCC97H (C) cells. Wound Healing assays were used to determine the cell capacity of migration for si-SRSF9 transfected HUH7 (B, I) and MHCC97H (D, J) cells. Apoptosis and cell cycle was determined by flow cytometry for si-SRSF9 mRNA transfected HUH7 (E, F, K, M) and MHCC97H (G, H, L, N) cells. Data were represented as the mean ± sem. From three independent experiments. *p < 0.05, **p < 0.01, ***p < 0.001, comparison with the si-NC group. [file 12943_2021_1493_MOESM5_ESM.docx]

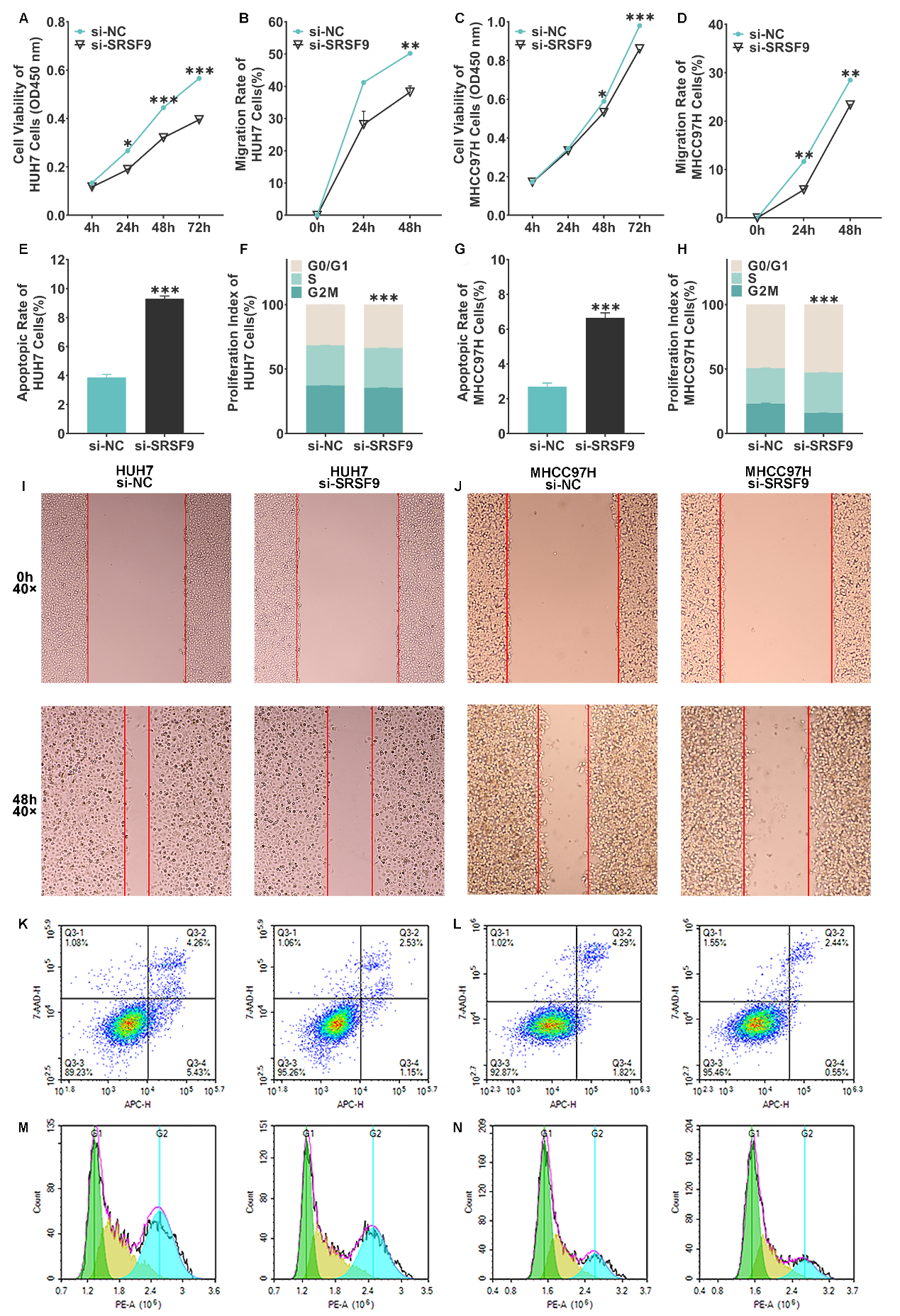


**Additional file 5: Figure S3**. **Suppression of SRSF9 mRNA significantly inhibits HCC cell proliferation, migration, cell cycle, and promotes HCC cell apoptosis.** CCK-8 assays were used to determine the cell viability for si-SRSF9 transfected HUH7 (A) and MHCC97H (C) cells. Wound Healing assays were used to determine the cell capacity of migration for si-SRSF9 transfected HUH7 (B, I) and MHCC97H (D, J) cells. Apoptosis and cell cycle was determined by flow cytometry for si-SRSF9 mRNA transfected HUH7 (E, F, K, M) and MHCC97H (G, H, L, N) cells. Data were represented as the mean ± sem. from three independent experiments. ^*^p < 0.05, ^**^p < 0.01, ^***^p < 0.001, comparison with the si-NC group.
