# Supplementary material for: Aberrant regulation of LncRNA TUG1-microRNA-328-3p-SRSF9 mRNA Axis in hepatocellular carcinoma: a promising target for prognosis and therapy
Source: Mol Cancer. 2022 Feb 4;21:36. doi: 10.1186/s12943-021-01493-6 (PMC8815183; doi:10.1186/s12943-021-01493-6)
Supplement: Supplementary file 6 — Additional file 6: Figure S4. Enforced expression of miR-328-3p efficiently inhibits HCC cell proliferation, migration, cell cycle, and promotes HCC cell apoptosis. CCK-8 assays were used to determine the cell viability for mimics-miR-328-3p transfected HUH7 (A) and MHCC97H (C) cells. Wound Healing assays were used to determine the cell ability of migration for mimics-miR-328-3p transfected HUH7 (B, I) and MHCC97H (D, J) cells. Apoptosis and cell cycle were determined by flow cytometry for mimics-miR-328-3p transfected HUH7 (E, F, K, M) and MHCC97H (G, H, L, N) cells. Data were represented as the mean ± sem. From three independent experiments. *p < 0.05, **p < 0.01, ***p < 0.001, comparison with the mimics-NC group. [file 12943_2021_1493_MOESM6_ESM.docx]

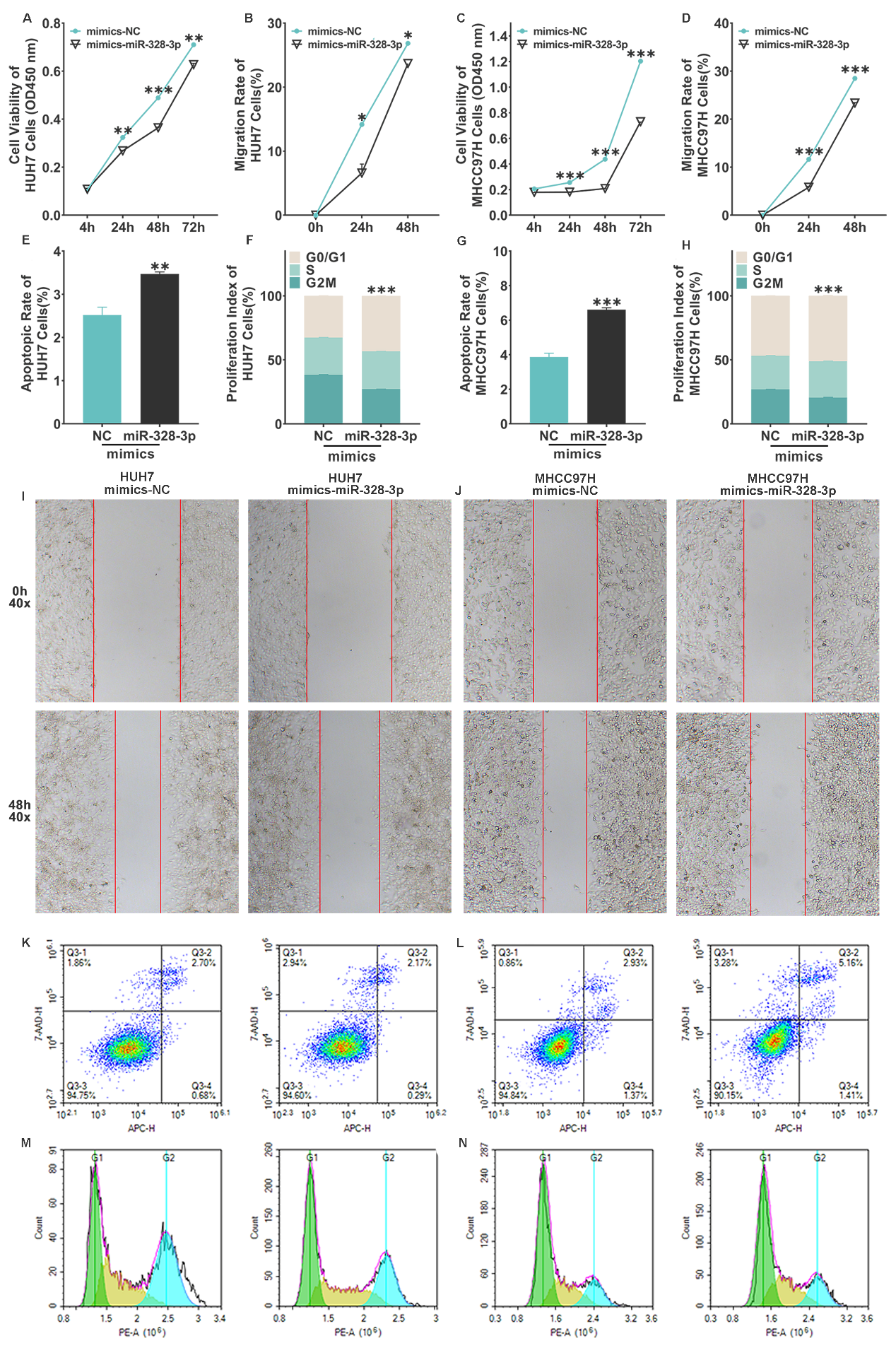


**Additional file 6: Figure S4.** **Enforced expression of miR-328-3p efficiently inhibits HCC cell proliferation, migration, cell cycle, and promotes HCC cell apoptosis. CCK-8 assays were used to determine the cell viability for mimics-miR-328-3p transfected HUH7 (A) and MHCC97H (C) cells. Wound Healing assays were used to determine the cell ability of migration for mimics-miR-328-3p transfected HUH7 (B, I) and MHCC97H (D, J) cells. Apoptosis and cell cycle were determined by flow cytometry for mimics-miR-328-3p transfected HUH7 (E, F, K, M) and MHCC97H (G, H, L, N) cells.** Data were represented as the mean ± sem. from three independent experiments. ^*^p < 0.05, ^**^p < 0.01, ^***^p < 0.001, comparison with the mimics-NC group.
