# Supplementary material for: Aberrant regulation of LncRNA TUG1-microRNA-328-3p-SRSF9 mRNA Axis in hepatocellular carcinoma: a promising target for prognosis and therapy
Source: Mol Cancer. 2022 Feb 4;21:36. doi: 10.1186/s12943-021-01493-6 (PMC8815183; doi:10.1186/s12943-021-01493-6)
Supplement: Supplementary file 7 — Additional file 7: Figure S5. The expression levels of SRSF9 protein in (A, B) HUH7, (C, D) MHCC97H cells that were imported with miR-328-3p and SRSF9 co-overexpression plasmids. Data were represented as the mean ± sem. From three independent experiments. *p < 0.05, **p < 0.01, ***p < 0.001, comparison with the pre-NC + mimics-NC group; &p < 0.05, &&p < 0.01, &&&p < 0.001, comparison with the pre-NC + mimics-miR-328-3p group. #p < 0.05, ##p < 0.01, ###p < 0.001, comparison with the pre-NC + pre-lncRNA TUG1/SRSF9 mRNA group. [file 12943_2021_1493_MOESM7_ESM.docx]

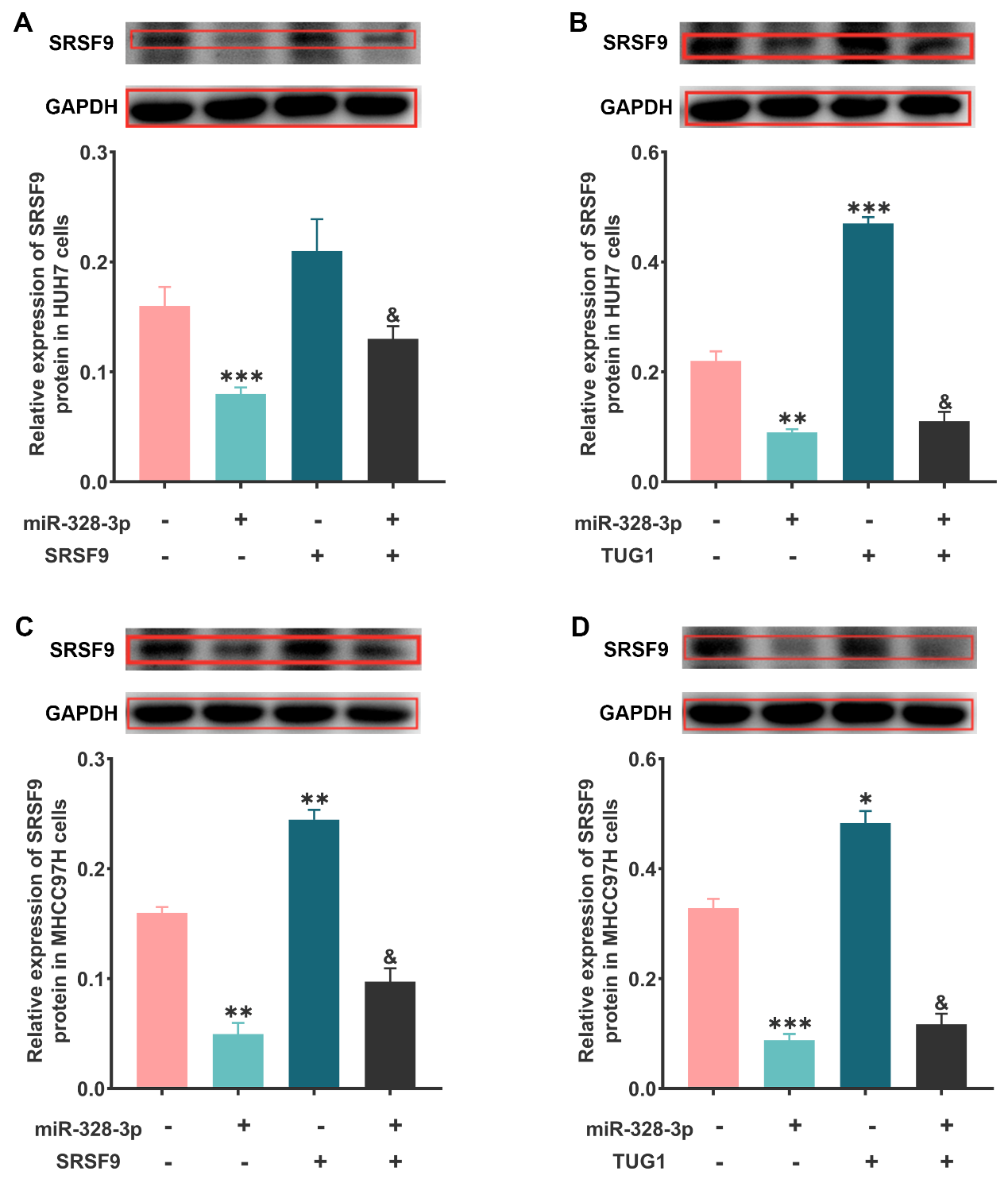


**Additional file 7: Figure S5. The expression levels of SRSF9 protein in (A, B) HUH7, (C, D) MHCC97H cells that were imported with miR-328-3p and SRSF9 co-overexpression plasmids.** Data were represented as the mean ± sem. from three independent experiments. ^*^p < 0.05, ^**^p < 0.01, ^***^p < 0.001, comparison with the pre-NC+mimics-NC group; ^&^p < 0.05, ^&&^p < 0.01, ^&&&^p < 0.001, comparison with the pre-NC+mimics-miR-328-3p group. ^#^p < 0.05, ^##^p < 0.01, ^###^p < 0.001, comparison with the pre-NC+pre-lncRNA TUG1/SRSF9 mRNA group.
