# Supplementary material for: Aberrant regulation of LncRNA TUG1-microRNA-328-3p-SRSF9 mRNA Axis in hepatocellular carcinoma: a promising target for prognosis and therapy
Source: Mol Cancer. 2022 Feb 4;21:36. doi: 10.1186/s12943-021-01493-6 (PMC8815183; doi:10.1186/s12943-021-01493-6)
Supplement: Supplementary file 8 — Additional file 8: Figure S6. Functional analysis of miR-328-3p alone, the co-transfection of miR-328-3p and SRSF9 mRNA, and the co-overexpression of miR-328-3p and lncRNA TUG1 in MHCC97H cells, analyzed via CCK-8 assay, wound healing assay and flow cytometry assay. A, B, D, E. SRSF9 overexpression reverses the tumor suppressive roles of miR-328-3p in the proliferation and migration activity in MHCC97H cells. CCK-8 assays were carried out to determine the cell viability for pre-SRSF9 mRNA+mimics-miR-328-3p transfected MHCC97H cells. Wound Healing assays were carried out to determine the cell mobility for pre-SRSF9 mRNA+mimics-miR-328-3p transfected MHCC97H cells. C, F, G. Apoptosis and cell cycle were determined by flow cytometry for pre-SRSF9 mRNA + mimics-miR-328-3p transfected MHCC97H cells. Data were represented as the mean ± sem. From three independent experiments. *p < 0.05, **p < 0.01, ***p < 0.001, comparison with the pre-NC + mimics-NC group; &p < 0.05, &&p < 0.01, &&&p < 0.001, comparison with the pre-NC + mimics-miR-328-3p group. #p < 0.05, ##p < 0.01, ###p < 0.001, comparison with the pre-NC + pre-SRSF9 group. H, I, K, L. LncRNA TUG1 competitively disturbs the regulatory effects of miR-328-3p in cell proliferation and migration in MHCC97H cells. CCK-8 assays were carried out to determine the cell viability for pre-lncRNA TUG1 + mimics-miR-328-3p transfected MHCC97H cells. Wound Healing assays were carried out to determine the cell mobility for pre-lncRNA TUG1 + mimics-miR-328-3p transfected MHCC97H cells. J, M, N. Apoptosis and cell cycle were determined by flow cytometry for pre-lncRNA TUG1 + mimics-miR-328-3p transfected MHCC97H cells. Data were represented as the mean ± sem. From three independent experiments. *p < 0.05, **p < 0.01, ***p < 0.001, comparison with the pre-NC + mimics-NC group; &p < 0.05, &&p < 0.01, &&&p < 0.001, comparison with the pre-NC + mimics-miR-328-3p group. #p < 0.05, ##p < 0.01, ###p < 0.001, comparison with the pre-NC + pre-l [file 12943_2021_1493_MOESM8_ESM.docx]

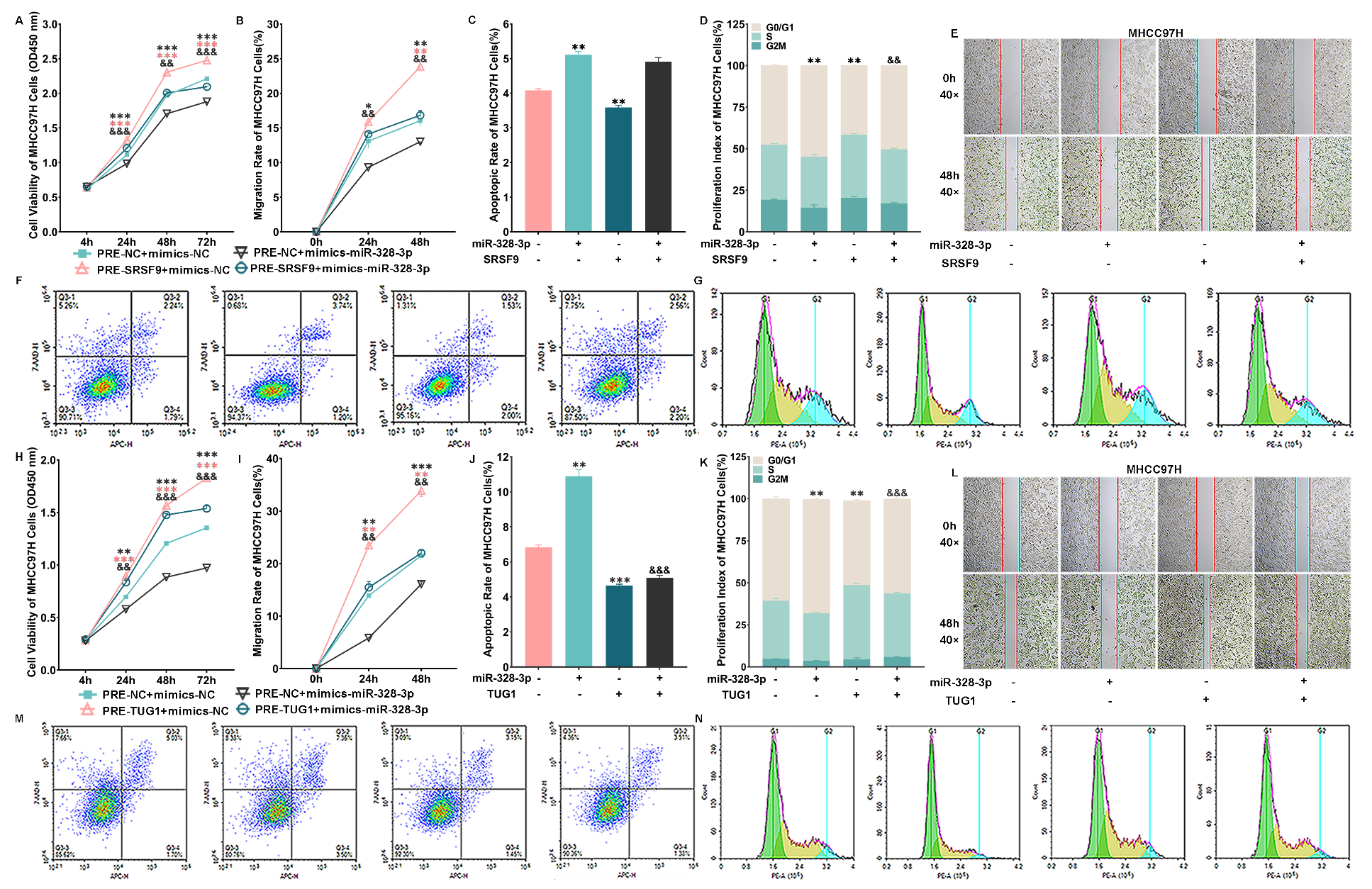


**Additional file 8: Figure S6. Functional analysis of miR-328-3p alone, the co-transfection of miR-328-3p and SRSF9 mRNA, and the co-overexpression of miR-328-3p and lncRNA TUG1 in MHCC97H cells, analyzed via CCK-8 assay, wound healing assay and flow cytometry assay. A, B, D, E. SRSF9 overexpression reverses the tumor suppressive roles of miR-328-3p in the proliferation and migration activity in MHCC97H cells.** CCK-8 assays were carried out to determine the cell viability for pre-SRSF9 mRNA+mimics-miR-328-3p transfected MHCC97H cells. Wound Healing assays were carried out to determine the cell mobility for pre-SRSF9 mRNA+mimics-miR-328-3p transfected MHCC97H cells. **C, F, G. Apoptosis and cell cycle were determined by flow cytometry for pre-SRSF9 mRNA+mimics-miR-328-3p transfected MHCC97H cells.** Data were represented as the mean ± sem. from three independent experiments. ^*^p < 0.05, ^**^p < 0.01, ^***^p < 0.001, comparison with the pre-NC+mimics-NC group; ^&^p < 0.05, ^&&^p < 0.01, ^&&&^p < 0.001, comparison with the pre-NC+mimics-miR-328-3p group. ^#^p < 0.05, ^##^p < 0.01, ^###^p < 0.001, comparison with the pre-NC+pre-SRSF9 group. **H, I, K, L. LncRNA TUG1 competitively disturbs the regulatory effects of miR-328-3p in cell proliferation and migration in MHCC97H cells.** CCK-8 assays were carried out to determine the cell viability for pre-lncRNA TUG1+mimics-miR-328-3p transfected MHCC97H cells. Wound Healing assays were carried out to determine the cell mobility for pre-lncRNA TUG1+mimics-miR-328-3p transfected MHCC97H cells. **J, M, N.** **Apoptosis and cell cycle were determined by flow cytometry for pre-lncRNA TUG1+mimics-miR-328-3p transfected MHCC97H cells.** Data were represented as the mean ± sem. from three independent experiments. ^*^p < 0.05, ^**^p < 0.01, ^***^p < 0.001, comparison with the pre-NC+mimics-NC group; ^&^p < 0.05, ^&&^p < 0.01, ^&&&^p < 0.001, comparison with the pre-NC+mimics-miR-328-3p group. ^#^p < 0.05, ^##^p < 0.01, ^###^p < 0.001, comparison with the pre-NC+pre-lncRNA TUG1 group.
