# Supplementary material for: Aberrant regulation of LncRNA TUG1-microRNA-328-3p-SRSF9 mRNA Axis in hepatocellular carcinoma: a promising target for prognosis and therapy
Source: Mol Cancer. 2022 Feb 4;21:36. doi: 10.1186/s12943-021-01493-6 (PMC8815183; doi:10.1186/s12943-021-01493-6)
Supplement: Supplementary file 9 — Additional file 9: Figure S7. Changes of the body weight of subcutaneous xenograft HCC nude mouse in control, model and FBRP treatment groups at different time points during experiments. *p < 0.05, **p < 0.01, ***p < 0.001, comparison with groups marked in corresponding color. [file 12943_2021_1493_MOESM9_ESM.docx]

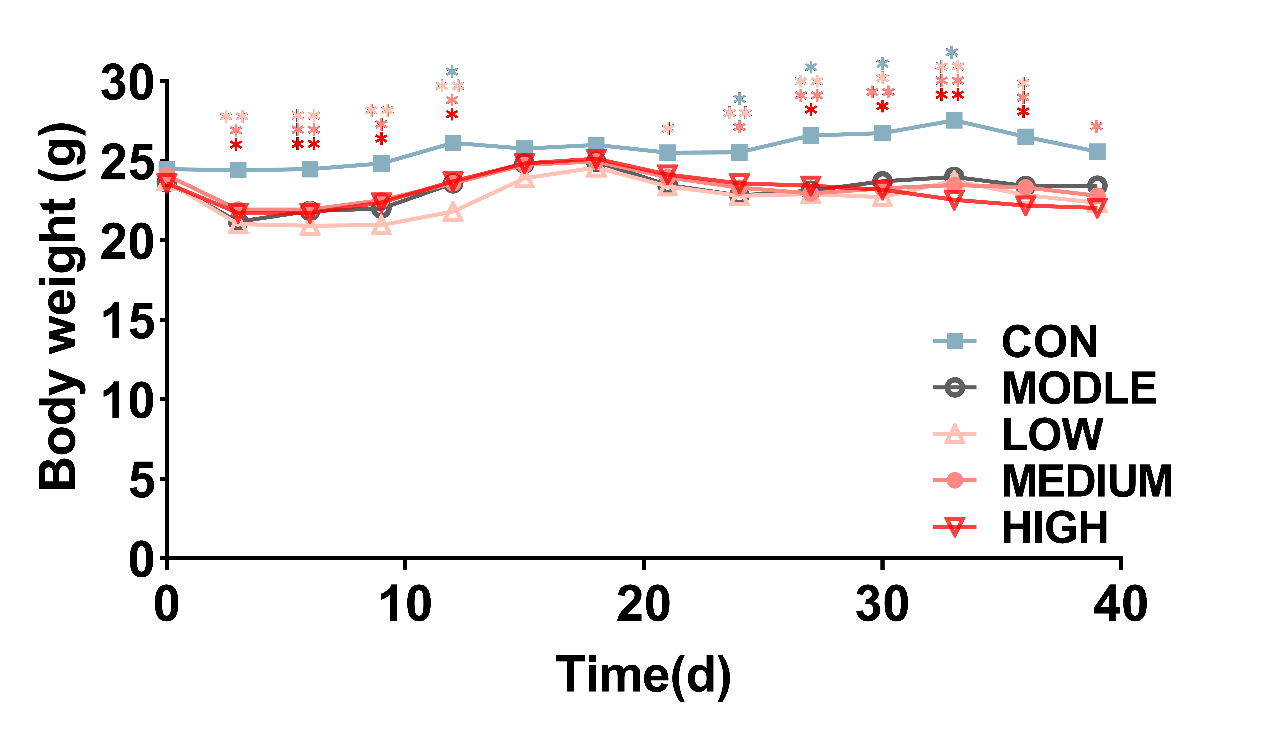
 **Additional file 9: Figure S7. Changes of the body weight of subcutaneous xenograft HCC nude mouse in control, model and FBRP treatment groups at different time points during experiments.** ^*^p < 0.05, ^**^p < 0.01, ^***^p < 0.001, comparison with groups marked in corresponding color.
