# Supplementary material for: Aberrant regulation of LncRNA TUG1-microRNA-328-3p-SRSF9 mRNA Axis in hepatocellular carcinoma: a promising target for prognosis and therapy
Source: Mol Cancer. 2022 Feb 4;21:36. doi: 10.1186/s12943-021-01493-6 (PMC8815183; doi:10.1186/s12943-021-01493-6)
Supplement: Supplementary file 10 — Additional file 10: Figure S8. Liver index of subcutaneous xenograft HCC nude mouse in control, model and FBRP treatment groups at different time points during experiments. [file 12943_2021_1493_MOESM10_ESM.docx]

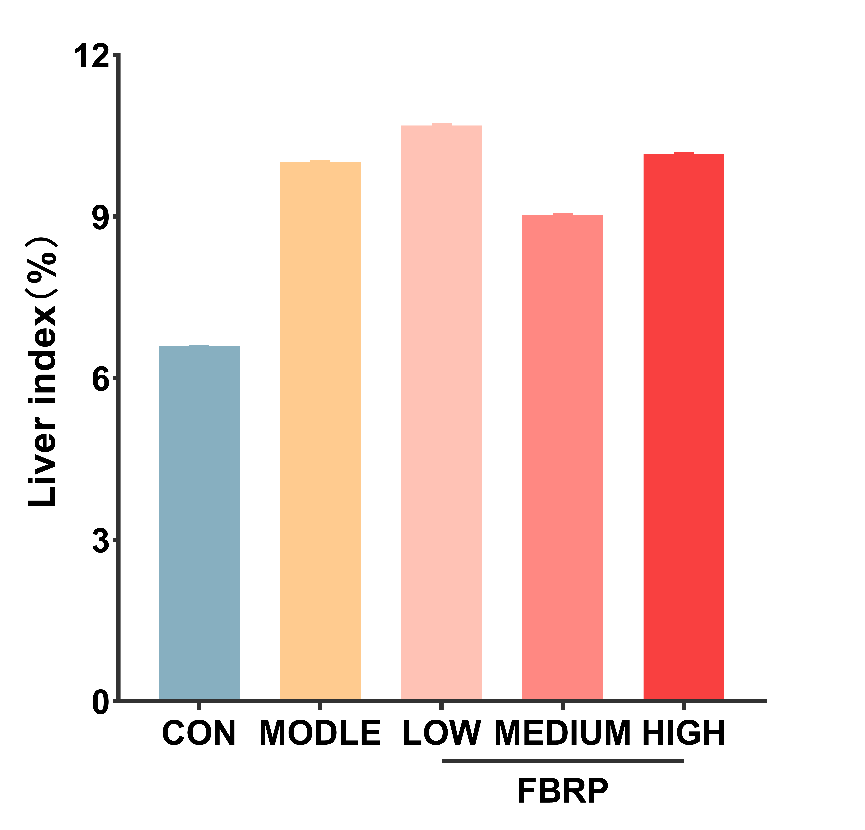


**Additional file 10: Figure S8. Liver index of subcutaneous xenograft HCC nude mouse in control, model and FBRP treatment groups at different time points during experiments.**
