# Supplementary material for: Aberrant regulation of LncRNA TUG1-microRNA-328-3p-SRSF9 mRNA Axis in hepatocellular carcinoma: a promising target for prognosis and therapy
Source: Mol Cancer. 2022 Feb 4;21:36. doi: 10.1186/s12943-021-01493-6 (PMC8815183; doi:10.1186/s12943-021-01493-6)
Supplement: Supplementary file 11 — Additional file 11. Materials and methods [file 12943_2021_1493_MOESM11_ESM.docx]

**Additional file 11: Materials and methods**

*Ethical statement*

This study was approved by the Ethics Committee of Institute of Chinese Materia Medica, China Academy of Chinese Medical Sciences, and the Fifth Medical Center of PLA General Hospital (No. 2016003D). All animal experiments were carried out in accordance with the guidelines and regulations for the care and use of laboratory animals of the Center for Laboratory Animal Care, China Academy of Chinese Medical Sciences, Beijing, China.

*Patients and specimens*

After obtaining the informed consent, a total of seventy-seven HCC tissues and fifty-six adjacent non-cancerous tissues were collected from HCC patients underwent hepatectomy between 2018.03~2019.09 at the Fifth Medical Center of PLA General Hospital (Beijing, China). Among them, three self-paired of fresh HCC and the corresponding adjacent non-cancerous liver tissues were used as the discovery cohort for microarray analysis, and the rest were used as the validation cohort for clinical validation on expression pattern of candidate RNAs. All tissues were pathologically confirmed and immediately frozen in liquid nitrogen. All HCC patients that received any embolotherapy or chemotherapy before surgical operation, and those accompanied with other organ tumors were excluded. 95% of patients enrolled in this study were diagnosed with HBV, and none of them were HCV-related. The clinicopathological and demographic information of the studied subjects were provided in **Table 1**. In addition, the publicly available dataset of HCC [Provisional, Tumor Samples with mRNA data (RNA Seq V2), 373 samples] collected from TCGA (https://www.cancer.gov/about-nci/organization/ccg/research/structural-genomics/tcga) was used to statistically analyze the correlation between identified mRNA/lncRNA and clinicopathological characteristics/prognosis of HCC patients. The detailed information of clinicopathological characteristics of HCC patients in our cohort and TCGA cohort, including patients' age, gender, preoperative serum AFP level, tumor stage, tumor grade, cirrhosis and invasion status were summarized in **Tables 2~3**.

**Table 1. Clinicopathological and demographic information of HCC patients in**

**discovery and validation cohorts enrolled in this study**

| **Features** | | **Discovery**  **Cohort（n=3）** | **Validation**  **Cohort（n=86）** |
| --- | --- | --- | --- |
| **Gender** | Male | 3 | 74 |
|  | Female | 0 | 12 |
| **Age (years)** | <50 | 3 | 42 |
|  | ≧50 | 0 | 44 |
| **Preoperative serum**  **AFP level**  **(Positive: higher than 400)** | Positive | 1 | 50 |
|  | Negative | 2 | 29 |
|  | Unknown | 0 | 7 |
| **Tumor stage** | T2 | 3 | 73 |
|  | T3 | 0 | 7 |
|  | Unknown | 0 | 6 |
| **Tumor grade** | G1-G2 | 0 | 70 |
|  | G3-G4 | 2 | 11 |
|  | Unknown | 1 | 5 |
| **Cirrhosis** | Positive | 3 | 71 |
|  | Negative | 0 | 14 |
|  | Unknown | 0 | 1 |
| **Tumors' Number** | Single | 3 | 72 |
|  | Multiple | 0 | 14 |

**Table 2. Associations of SRSF9 and lncRNA TUG1 expression and various clinicopathological features in**

**HCC patients of our cohort**

| **Features** | | **SRSF9 expression** | | ***P*** | **TUG1 expression** | | ***P*** |
| --- | --- | --- | --- | --- | --- | --- | --- |
|  |  | **High(n)** | **Low(n)** |  | **High(n)** | **Low(n)** |  |
| **Gender** | Male | 34 | 33 | >0.05 | 34 | 33 | >0.05 |
|  | Female | 5 | 5 |  | 5 | 5 |  |
| **Age (years)** | ≧50 | 21 | 17 | >0.05 | 19 | 19 | >0.05 |
|  | <50 | 18 | 21 |  | 20 | 19 |  |
| **Preoperative serum AFP level** | ≧20 | 13 | 22 | 0.01<p<0.05 | 13 | 20 | 0.01<p<0.05 |
|  | <20 | 25 | 15 |  | 26 | 14 |  |
| **Tumor stage** | T2 | 35 | 31 | >0.05 | 36 | 30 | >0.05 |
|  | T3 | 2 | 3 |  | 1 | 4 |  |
| **Tumor grade** | G2 | 31 | 25 | >0.05 | 32 | 24 | >0.05 |
|  | G3 | 7 | 9 |  | 5 | 11 |  |
| **Cirrhosis** | Positive | 28 | 34 | 0.01<p<0.05 | 28 | 34 | 0.01<p<0.05 |
|  | Negative | 11 | 3 |  | 11 | 3 |  |

**Table 3. Associations of SRSF9 and lncRNA TUG1 expression and various clinicopathological features in**

**HCC patients of TCGA cohort**

| **Features** | | **SRSF9 expression** | | ***P*** | **TUG1 expression** | | ***P*** |
| --- | --- | --- | --- | --- | --- | --- | --- |
|  |  | **High（n）** | **Low（n）** |  | **High（n）** | **Low（n）** |  |
| **Gender** | Male | 132 | 120 | 0.01<p<0.05 | 102 | 144 | <0.01 |
|  | Female | 55 | 66 |  | 81 | 39 |  |
| **Age (years)** | >61 | 96 | 81 | >0.05 | 84 | 91 | >0.05 |
|  | ≤61 | 90 | 105 |  | 99 | 91 |  |
| **Preoperative serum AFP level** | Positive | 92 | 57 | <0.01 | 69 | 103 | <0.01 |
|  | Negative | 45 | 85 |  | 64 | 38 |  |
| **Tumor Stage (American Joint Committee on Cancer)** | T1-T2 | 134 | 143 | 0.01<p<0.05 | 126 | 146 | 0.01<p<0.05 |
|  | T3-T4 | 53 | 40 |  | 56 | 36 |  |
| **Disease Free Status** | Non- Recurred | 71 | 74 | 0.03 | 82 | 91 | 0.09 |
|  | Recurred | 90 | 86 |  | 71 | 70 |  |
| **Overall Survival Status** | Living | 114 | 129 | 0.03 | 113 | 124 | 0.31 |
|  | Deceased | 73 | 57 |  | 70 | 59 |  |
| **Liver fibrosis ishak score** | 0 (No Fibrosis) | 26 | 49 | 0.01<p<0.05 | 38 | 36 | >0.05 |
|  | 1～4 （Portal Fibrosis~ Fibrous Speta） | 29 | 30 |  | 26 | 32 |  |
|  | 5~6 （Nodular Formation and Incomplete Cirrhosis Established Cirrhosis） | 45 | 34 |  | 32 | 45 |  |
| **Neoplasm Disease Stage (American Joint Committee on Cancer)** | Stage I | 82 | 90 | >0.05 | 78 | 91 | >0.05 |
|  | Stage II | 44 | 42 |  | 38 | 48 |  |
|  | Stage III | 46 | 39 |  | 52 | 32 |  |
|  | Stage IV | 3 | 2 |  | 3 | 3 |  |
| **Neoplasm Histologic Grade** | G1 | 22 | 33 | 0.01<p<0.05 | 19 | 35 | >0.05 |
|  | G2 | 83 | 95 |  | 85 | 88 |  |
|  | G3 | 71 | 52 |  | 68 | 54 |  |
|  | G4 | 9 | 3 |  | 7 | 5 |  |
| **Vascular Invasion** | Absent | 102 | 105 | >0.05 | 102 | 99 | >0.05 |
|  | Present | 55 | 54 |  | 55 | 59 |  |

*Cell culture*

Human HCC cell lines (HUH7 and MHCC97H) and 293T cells were obtained from Cell Biology of the Chinese Academy of Sciences (Shanghai, China). Dulbecco's Modified Eagle Medium (DMEM) High Glucose (Hyclone, Illinois, USA) medium supplemented with 10% heat-inactivated FBS, 100U/ml penicillin and 100 mg/ml streptomycin (GIBCO, CA, USA) were applied to maintain the cells at 37°C with 5% CO_2_. HUH7 and MHCC97H were selected in this study due to their high-metastatic capacity, which benefits us to explore the effects of SRSF9 on cell migration capacity by cell functional experiments ^[1]^. In addition, the two cells can be subcutaneously injected into rats or nude mice to construct xenografted tumor model and *in situ* model in nude mice with high success ratio for *in vivo* HCC experiments ^[2]^.

*Microarray-based gene expression profiling*

Microarray analysis was performed to detect gene expression profiling of human HCC and adjacent non-cancerous liver tissues. Sample preparation, microarray hybridization, labeling, scan and analysis were carried out by Shanghai GMINIX Biotechnology Corporation (Shanghai, China). The expression profiles of miRNA and mRNA/lncRNA were identified by Affymetrix GeneChip miRNA 2.0 Arrays (Thermo Fisher Scientific, MA, CA) and Affymetrix Clariom D Human array (Thermo Fisher Scientific, MA, CA), respectively.

*Prediction of miRNA targets*

The candidate putative lncRNAs and mRNAs for the selected miRNA were predicted using miRanda (http://www.microrna.org/microrna/home.do) and TargetScan (http://www.targetscan.org/vert_72/). In combination with the lncRNA-miRNA, mRNA-miRNA interaction that target the same 3′‐untranslated region (3′‐UTR) sites, the candidate lncRNA-miRNA-mRNA axis was identified.

*Real-time quantative PCR (Real-time qPCR)*

The total RNA of tissues and cells were extracted using Trizol reagent (Invitrogen, Carlsbad, CA) according to manufacturer’s instructions. The reverse transcription of lncRNA and mRNA was completed using RevertAid First Strand cDNA Synthesis Kit (Thermo Fisher Scientific, MA, CA), and real-time qPCR analysis using a UltraSYBR Mixture (High ROX) (BeiJing Cowin Biotech, Beijing, China). Meanwhile, the reverse transcription of miRNA used TB Green® Premix Ex Taq™ II (Tli RNaseH Plus) (Takara Biomedical Technology, Oshijin City, Japan), and RT-PCR analysis was completed by TB Green Advantage Real-time PCR Premix (Takara Biomedical Technology, Oshijin City, Japan). DDX5 and U6 was used as an endogenous control for detecting the expression of lncRNA/mRNA and miRNA. The relative expression levels were calculated by using the comparative CT (2^-ΔΔCT^) method. Each assay was repeated three times independently of each other. A list of primers used for real-time qPCR experiments were included in **Table 4**.

**Table 4. Detailed information of primers used for real-time qPCR experiments**

**in this study**

| **Species** | **RNA Name** | **Sequence: 5’-3’** |
| --- | --- | --- |
| Homo sapiens | DDX5 | F:CTTGTCCTTGATGAAGCAGA |
|  |  | R:AGTCGCACTCCACATTAG |
|  | SRSF9 | F:TCGGCTTCGTGTGGAGTTC |
|  |  | R:AGCTTCTCGCATGTGATCCTTC |
|  | TUG1 | F:CTGAAGAAAGGCAACATC |
|  |  | R:GTAGGCTACTACAGGATTTG |
|  | U6 | F:GCTTCGGCAGCACATATACTAAAAT |
|  |  | R:CGCTTCAGAATTTGCGTGTCAT |
|  | miR-328-3p | CTGGCCCTCTCTGCCCTT |
| Rattus norvegicus | β-Actin | F:AAGATCAAGATCATTGCTCCTCCT |
|  |  | R:AGCTCAGTAACAGTCCGCCT |
|  | SRSF9 | F:GCAGACGTACAGAAGGACGG |
|  |  | R:ACACCCGGATGTACGAAGTC |
|  | TUG1 | F:TGCACTGGGTAAACGTTGGA |
|  |  | R:TAACTGGGTATGGCAGGAGGG |
|  | U6 | GCAAATTCGTGAAGCGTTCC |
|  | miR-328-3p | GCCCTCTCTGCCCTTCCGT |

*Dual-luciferase reporter gene assay*

The wild-type and mutant fragments in 3′‐UTR of lncRNA TUG1 and SRSF9 mRNA related to the miR-328-3P binding site were synthesized and inserted into psiCHECK-2 vectors (Promega, Madison, USA), then mixed with miR-328-3P mimics and co-transfected to 293T cell, respectively. After transfection for 48h, fluorescence detection was performed using dual-luciferase reporter gene detection kit (LF005, GeneCopoeia, Guangzhou, China) and the relative luciferase activity was calculated.

*Plasmids construction and cell transfection*

For overexpressing SRSF9 mRNA, one lentivector-mediated short hairpin SRSF9 mRNA (NM_003769.3, pLVSO2-SRSF9) and non-targeting plasmids (pLVSO2) were designed and synthesized. Lentivirus infection of HCC cells was performed. The full-length of human TUG1 cDNA was synthesized and sub cloned into a pCDNA3.1 (Invitrogen, CA, USA) vector (NR_002323). The empty plasmid pcDNA3.1 was used as negative control (EV). miR-328-3P mimics and negative control mimics were obtained from Guangzhou Genaral Co., Ltd. (Guangzhou, China), and a small interfering RNA (siRNA) targeting SRSF9 mRNA (GGTCGAGTATCTCAGAAAA) and scrambled siRNA obtained from Guangzhou RiboBio Co., Ltd. (Guangzhou, China). Plasmids were transfected into cells using Lipofectamine 2000 (Invitrogen) following the manufacturer’s protocol. The transfection efficiency was confirmed by western blot and RT-PCR analysis.

*Cell counting Kit-8 (CCK-8) assay*

HCC cell suspensions were added to a 96-well plate at an initial density of 1×10^4^/mL. The cells were stained with 10μL of CCK-8 solution (Beyotime Biotechnology, Jiangsu, China) for 4h in incubator in the dark. Finally, the absorbance at 450nm was measured using a microplate reader (Thermo Fisher Scientific, MA, CA) after culturing for 0h, 24h, 48h and 72h. All experiments were carried out in triplicate.

*Flow cytometry assay*

HCC cells in different groups were seeded and treated as indicated. For cell apoptosis assay, the Annexin V-FITC Apoptosis Detection Kit [MultiSciences (LiankeBio), Hangzhou, China] was used following the manufacturer’s protocol. After washing twice with pre-cold PBS buffer, cells were stained with PI and Annexin-V for 15min at room temperature in the dark. For cell cycle, cells were collected and fixed using 70% ethanol. Cells were stained with stain buffer after washing with PBS and then 1×10^6^ cells were resuspended in 0.5mL of PI/RNase Staining Buffer (BD Biosciences, New Jersey, USA), and incubated for 15min at room temperature protected from light. Flow cytometry (BD Biosciences, New Jersey, USA) was used to analyze cell apoptosis and cycle phase distribution with cellular DNA contents.

*Wound healing assay*

Appropriate cell density was required to attain 90% confluence for HCC cells after 24h of transfection of 6-well plates. A straight scratch was formed in the center of each well using a pipette tip to perpendicular scrape cells after adding 4μg/mL mitomycin (Sigma-Aldrich, CA, USA). Cells were observed at different time points of 0h, 24h and 48h, and assessed by measuring the movement distance of the cells into the scratch per well.

*Western blot*

Proteins in HCC cells were lysed with RIPA buffer (Beyotime, Shanghai, China) and quantified using a BCA protein assay kit II (BIO-RAD, Hercules, CA, USA). Then, protein samples were separated by 10% SDS-PAGE gel and transferred into PVDF membranes, which were incubated with primary antibody against SFSR9 (ab236414, Abcam, MA, USA) and GAPDH (10494-1-AP, Proteintech, Chicago, USA) overnight at 4°C. After washing, horseradish peroxidase (HRP)-conjugated secondary antibodies (ab205719, Abcam, MA, USA) was applied to incubate membranes for 2h at room temperature. An ECL detection system (Bio-Rad, USA) was used to detect the blot signals.

*Animals*

Animals were raised under specific pathogen-free conditions with the temperature (22±2ºC) and humidity (55±5%), and a 12 hours light-dark cycle. They were allowed free access to food and water *ad libitum* during experiments.

*Nude mice xenograft*

A total of 37 male BLAB/c mice aged 4~6 weeks were purchased from Guangdong Medical Laboratory Animal Center (SCXK-2013-0002). About 3.0×10^6^ Huh7 cells/0.05mL single cell suspension were inoculated into the liver lobe of nude mice. The mice were randomly divided into five groups, including normal control group (n=5), model group (n=8), and low, middle high-dose FBRP treatment group (n=8,8,8) (0.25g/kg, 0.5g/kg and 1.0g/kg mice weight FBRP, administrated by gavage every day). These three dosages selection for FBRP was 0.5, 1 and 2 times of the daily dosage of hepatic fibrosis patients in clinics, respectively. When the body weight began to loss, which were measured every three days, the nude mice were sacrificed using cervical dislocation, and liver tissues sectionally fixed in 10% formalin, and the remaining portions immediately stored at -80ºC. The size of the tumor nodules was measured.

*Chronic inflammation-associated HCC model*

To induce hepatocarcinogenesis, male Sprague-Dawley rats (n=26) weighing 240g~260g were purchased from Guangdong Medical Laboratory Animal Center (SCXK 2013-0002) and randomly divided into four groups, including normal control group (n=3), diethylnitrosamine (DEN)-induced model group (n=6), FBRP group (n=9) and DEN-FBRP treatment group (n=9). Briefly, rats in the DEN model and DEN-FBRP treatment groups were intragastrical administrated 0.01% DEN (Sigma-Aldrich, St. Louis, MO, USA) for a period of 18 weeks, the latter were given 1.0g/kg FBRP (Furui Medical Science, Beijing, China) at the same time. Rats in normal control group were administrated with the same volume of saline and in FBRP group received daily oral administration of 1.0g/kg FBRP, which was twice the daily dosage of hepatic fibrosis patients in clinics and exerted the most prominent therapeutic efficacy in patients with hepatic fibrosis.

Pharmacodynamic indicators including body weight, tumor size, liver index, the ratio of liver weight to body weight were measured and analyzed has been described in our previous studies ^[12]^. After sacrifice, the hepatic tissues were dissected out, weighed and sectionally fixed in 10% formalin, and the remaining portions immediately stored at -80ºC until use.

*Histological analysis*

The hepatic tissues from nude mice were immobilized and embedded in 10% formalin buffer, then sectioned at the thickness of 4µm. Representative fragments were dewaxed, stained in hematoxylin and 0.5% eosin-phloxine solution for 5min and 3min in sequence, and fixed with neutral resin after dehydration for microscopic observations.

*Immunohistochemistry*

Immunostaining was performed to determine the subcellular localization and expression pattern of SRSF9 protein in HCC tissues from nude mice. Briefly, paraffin-embedded samples were cut into a thickness of 4µm, and then deparaffinized, hydrated, incubated with 0.3% hydrogen peroxide for 15 mins for blocking endogenous peroxidase, and heat antigen-repaired by microwave. Sections were incubated with anti-SRSF9 mouse monoclonal antibody (1:150, ab236414, Abcam, MA, USA) at 4°C overnight. After washing with phosphate buffer saline (PBS), sections were incubated with secondary biotinylated goat anti-mouse IgG for 30 mins at 37°C, and stained with 3,3-diaminobenzidin and hematoxylin solution, which were subsequently sealed with neutral resins. Finally, the images were photographed, and then analyzed by Image-Pro Plus 6.0 software.

*Fluorescence in situ hybridization (FISH)*

The FISH assay was performed in HCC tissues from nude mice according to the specifications of the manufacturers. The Cy3-labeled TUG1 probe (GUGAGUCGUGUCUCUCUUUUCUC) and FITC-labeled miR-328-3P probe (ACGGAAGGGCAGAGAGGGCCAG) used in this study were designed and synthesized by GENETAL BIO company (Anhui, China). The prepared HCC tissues were fixed in 4% paraformaldehyde for 30 min, and incubated with specific probes at 37°C overnight after permeabilization. The cell nuclei were stained with DAPI (Sigma-Aldrich, CA, USA) and observed using a fluorescence microscope (Nikon, Tokyo, Japan).

*Statistical analyses*

GraphPad prism 5.0 software (GraphPad Software, CA, USA) was used to analyze data for statistical significance. Chi-squared test was applied to evaluate the associations between SRSF9 mRNA expression and various clinicopathological characteristics of HCC patients. Disease free status and overall survival status were plotted by Kaplan-Meier method. Two-tailed Student’s t-test was used for comparing difference between two independent groups. A value of two-tailed P<0.05 is considered as statistically significant.

**References**

1. Yang B, Feng X, Liu H, Tong R, Wu J, Li C, Yu H, Chen Y, Cheng Q, Chen J, Cai X, Wu W, Lu Y, Hu J, Liang K, Lv Z, Wu J, Zheng S. High-metastatic cancer cells derived exosomal miR92a-3p promotes epithelial-mesenchymal transition and metastasis of low-metastatic cancer cells by regulating PTEN/Akt pathway in hepatocellular carcinoma. Oncogene. 2020 Oct;39(42):6529-6543. doi: 10.1038/s41388-020-01450-5.
2. Cunhua Shao, Gongpan Liu, Xiaobin Zhang, Anyun Li, Xingjun Guo. Long Noncoding RNA RMRP Suppresses the Tumorigenesis of Hepatocellular Carcinoma Through Targeting microRNA-766. Onco Targets Ther. 2020 Apr 8;13:3013-3024. doi: 10.2147/OTT.S243736.
